# The human protein disulfide isomerase gene family

**DOI:** 10.1186/1479-7364-6-6

**Published:** 2012-07-05

**Authors:** James J Galligan, Dennis R Petersen

**Affiliations:** 1 Department of Pharmacology, University of Colorado Anschutz Medical Campus, Aurora, CO, 80045, USA; 2 Molecular Toxicology and Environmental Health Sciences Program, Department of Pharmacy and Pharmaceutical Sciences, University of Colorado Anschutz Medical Campus, Aurora, CO, 80045, USA

**Keywords:** Disulfide bond, Thioredoxin, Calsequestrin, UPR, Unfolded protein response, ER stress

## Abstract

Enzyme-mediated disulfide bond formation is a highly conserved process affecting over one-third of all eukaryotic proteins. The enzymes primarily responsible for facilitating thiol-disulfide exchange are members of an expanding family of proteins known as protein disulfide isomerases (PDIs). These proteins are part of a larger superfamily of proteins known as the thioredoxin protein family (TRX). As members of the PDI family of proteins, all proteins contain a TRX-like structural domain and are predominantly expressed in the endoplasmic reticulum. Subcellular localization and the presence of a TRX domain, however, comprise the short list of distinguishing features required for gene family classification. To date, the *PDI* gene family contains 21 members, varying in domain composition, molecular weight, tissue expression, and cellular processing. Given their vital role in protein-folding, loss of PDI activity has been associated with the pathogenesis of numerous disease states, most commonly related to the unfolded protein response (UPR). Over the past decade, UPR has become a very attractive therapeutic target for multiple pathologies including Alzheimer disease, Parkinson disease, alcoholic and non-alcoholic liver disease, and type-2 diabetes. Understanding the mechanisms of protein-folding, specifically thiol-disulfide exchange, may lead to development of a novel class of therapeutics that would help alleviate a wide range of diseases by targeting the UPR.

## Introduction

Increasing evidence supports an important role for misfolded proteins in the pathogenesis of numerous diseases including diabetes, Alzheimer disease, Parkinson disease, and both alcoholic and non-alcoholic liver disease [1]. Accumulation of misfolded proteins (or erred protein load) is generally caused by either decreased disposal of erred protein or a decrease in the correct folding of proteins [1]. Disulfide bond formation represents a fundamentally important post-translational modification that is a critical step in the folding of nascent peptides in the endoplasmic reticulum (ER) [2]. These covalent linkages are formed between the side-chains of cysteine residues and represent a key rate-limiting step in protein maturation [3]. The enzymatic formation, breakage, and subsequent rearrangement of cysteine linkages are crucial to protein structure and function and primarily mediated by members of the protein disulfide isomerase (PDI) family [4]. All genes in the *PDI* family are part of a superfamily referred to as the thioredoxin (TRX) superfamily, which also includes the glutaredoxins, TRXs, ferroredoxins, and peroxidoxins [5].

The *PDI* gene family currently comprises 21 genes, varying in size, expression, localization, and enzymatic function. Although it is implied that all members of the PDI family possess the ability to rearrange disulfide bonds, only a subset is considered orthologous and able to carry out these reactions, with the other members being paralogous and linked to the family through evolution, not function [4]. While these proteins may be functionally different, the unifying feature of all PDI family members is the presence of a TRX-like domain [2]. These may be present as either a catalytically active **a** or **a’** domain (the presence of a CXXC motif) or a catalytically inactive **b** or **b’** domain (for a more detailed review on the precise role of these domains, see the work of Ellgaard et al.) [2,4]. Extensive research has assessed the roles of these domains and revealed the **b’** domain to be the primary peptide- or protein-binding domain [4]. Previous literature has highlighted the features of a number of *PDI* family members; however, with an increasing amount of cDNA and EST sequence information deposited in the NCBI database, a composite review is required to further characterize and compare all 21 current members of the *PDI* gene family (Table 1).

**Table 1:** **Human *PDI* genes as listed in the Human Gene Nomenclature Committee (HGNC) database**

**Gene name**	**Other aliases**	**Protein name**	**Chromosomal location**	**Amino acids**
*AGR2*	XAG-2, HAG-2, AG2, PDIA17	Anterior gradient protein 2 homolog	7p21.3	175
*AGR3*	HAG3, hAG-3, BCMP11, PDIA18	Anterior gradient protein 3 homolog	7p21.1	166
*CASQ1*	PDIB1	Calsequestrin-1	1q21	396
*CASQ2*	PDIB2	Calsequestrin-2	1p13.3-p11	399
*DNAJC10*	MTHr, ERdj5	DnaJ (Hsp40) homolog, subfamily C, member 10	2q32.1	793
*ERP27*	FLJ32115, ERp27, PDIA8	Endoplasmic reticulum resident protein 27	12p12.3	273
*ERP29*	ERp28, ERp31, ERp29, PDI-DB, PDIA9	Endoplasmic reticulum resident protein 29	12q24.13	261
*ERP44*	KIAA0573, PDIA10	Endoplasmic reticulum resident protein 44	9q22.33	406
*P4HB*	DIA1, PROHB, DSI, GIT, PDI, PO4HB, P4Hb	Protein disulfide-isomerase	17q25	508
*PDIA2*	PDA2, PDIp	Protein disulfide-isomerase A2	16p13.3	525
*PDIA3*	P58, ERp61, ERp57, ERp60, GRP57, PI-PLC, HsT17083	Protein disulfide-isomerase A3	15q15	505
*PDIA4*	ERP70, ERP72	Protein disulfide-isomerase A4	7q35	645
*PDIA5*	PDIR, FLJ30401	Protein disulfide-isomerase A5	3q21.1	519
*PDIA6*	P5, ERp5	Protein disulfide-isomerase A6	2p25.1	440
*PDILT*	PDIA7, ERp65	Protein disulfide-isomerase-like protein of the testis	16p12.3	584
*TMX1*	TMX, PDIA11	Thioredoxin-related transmembrane protein 1	14q22.1	280
*TMX2*	PDIA12	Thioredoxin-related transmembrane protein 2	11cen-q22.3	296
*TMX3*	FLJ20793, KIAA1830, PDIA13	Protein disulfide-isomerase TMX3	18q22	454
*TMX4*	DJ971N18.2, KIAA1162, PDIA14	Thioredoxin-related transmembrane protein 4	20p12	349
*TXNDC5*	MGC3178, FLJ21353, FLJ90810, EndoPDI, Hcc-2, ERp46, PDIA15	Thioredoxin domain-containing protein 5	6p24.3	432
*TXNDC12*	TLP19, ERP18, ERP19, hAG-1, AGR1, PDIA16	Thioredoxin domain-containing protein 12	1p32.3	172

*DNAJC10* has been included following identification of this gene in the *PDI* gene family. Synonyms of these genes have also been corrected.

## Domain composition of the PDI family proteins

**Figure 1:**
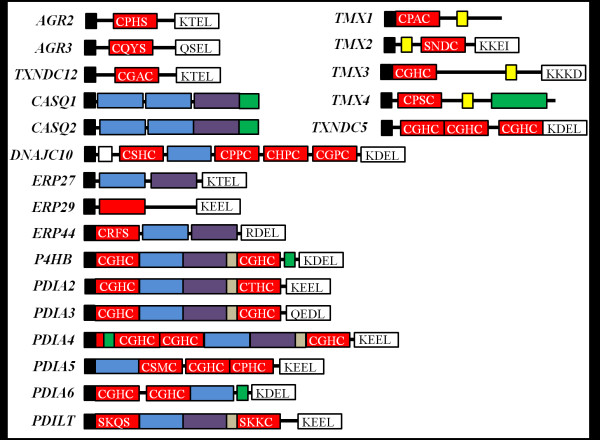
**Schematic representation of the domain compositions of the 21 proteins in the *PDI* gene family.** All proteins contain a short NH_2_-terminal signal sequence designated in black. Catalytically active TRX-like domains (**a** or **a’**) are represented in *red* with active sites noted; inactive TRX domains in *blue* (**b**) and *purple* (**b’**); *green* represents the Asp/Glu rich Ca^2+^-binding domains; linker regions (*gray*); transmembrane domains (*yellow*); COOH-terminal ER-retention sequences in *white* with a.a. composition denoted. Although ERP29 does contain an **a-**like domain, this is based on homology and not catalytic efficiency. Sequences and domain compositions were acquired and verified from the National Center for Biotechnology Information (NCBI) database. Figure was adapted and modified from [4].

Proteins in the PDI family are largely expressed in the ER, although few family members have been detected in other subcellular compartments [6]. Due to their localization, the presence of a short NH_2_-terminal signal peptide exists in all members of the family. These peptides are typically 15–30 amino acids (a.a.) in length and are cleaved upon entry into the ER [7]; this has led to some sequence discrepancy among multiple PDI proteins. As indicated, the common thread between all members of the PDI proteins is the presence of at least one TRX-like domain, whether it is catalytically active (**a**) or inactive (**b**) [2]. The active site of the **a**-type domains also varies greatly, with the “classical” motif being comprised of Cys-Gly-His-Cys. The cysteine residues in these active sites are considered redox active, undergoing active shuffling of disulfide bonds [2]. The surrounding a.a. largely play a role in the regulation of the pKa of the cysteines, dictating the local redox potential and thus regulating the catalytic ability of these cysteines to actively oxidize or reduce disulfide bonds (for a more comprehensive review on the redox potential of PDI, see the work of Hatahet et al*.*) [3]. Extensive biochemical and biophysical experimentation has taken place analyzing TRX–like domain containing proteins; however, a complete crystal structure is currently not available for most family members. Another common characteristic of the PDI family of proteins is the presence of a COOH-terminal ER retention sequence comprised of a Lys-Asp-Glu-Leu-like sequence [2]. Whereas these sequences may differ greatly in a.a. composition, only four PDI proteins do not contain this sequence. Figure 1 shows the domain composition of the 21 proteins in the PDI family.

## Evolutionary divergence of the *PDI* gene family

**Figure 2:**
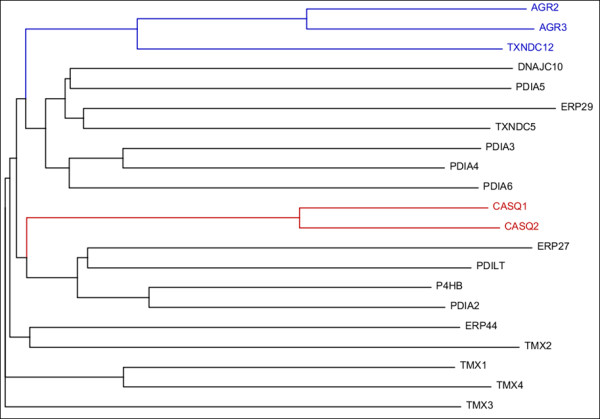
**Clustering dendrogram of the human *PDI* gene family.** Utilizing ClustalW alignment software of known protein sequences of the human *PDI* genes reveals divergence into *AGR* and *CASQ* subfamilies (red and blue, respectively).

As mentioned, all genes encompassed in the *PDI* family belong to the *TRX* superfamily of genes [5]. The unifying theme between these proteins is the presence of at least one TRX-like domain, whether this be catalytically active (**a or a’**) or inactive (**b** or **b’**) [4]. These domains contain a TRX structural fold that has amino acids arranged in a conserved three-dimensional conformation [8]. While the enzymatic function of these domains is not conserved, the current theory proposes that all PDI family members evolved through domain duplications from an ancestral prokaryotic PDI which contained a single TRX domain [9]. Although all human PDIs possess a TRX-like domain, this remains one of the few similarities between these proteins, differing greatly in molecular mass and a.a. composition outside of the TRX fold. Phylogenetic analysis, presented as a dendrogram in Figure 2, outlines the evolutionary divergence of the human PDI proteins. Sequence analysis reveals a subset of genes within this family that are evolutionarily related, as shown by the calsequestrin (CASQ) and anterior gradient (AGR) branches (in red and blue, respectively). Supporting the hypothesis that these subsets of genes are phylogenetically related, a high degree of similarity was also observed with both domain architecture (Figure 1) and sequence identity (Table 2). Although similarities are evident, the overall homology between the proteins is quite minimal. This is supported by previous attempts to cluster large sets of both eukaryotic and prokaryotic PDIs where marginal resolution of PDI domains was also observed [9]. Given the broad spectrum of both enzymatic functions and domain compositions, it is not surprising that the proteins share little sequence homology with one another (Table 2).

**Table 2:** **Amino acid similarity between human PDI proteins, as reported using EMBOSS Water Pairwise Alignment Algorithm (**http://www.ebi.ac.uk/Tools/psa/emboss_water/**)**

	**AGR2**	**AGR3**	**CASQ1**	**CASQ2**	**DNAJC10**	**ERP27**	**ERP29**	**ERP44**	**P4HB**	**PDIA2**	**PDIA3**	**PDIA4**	**PDIA5**	**PDIA6**	**PDILT**	**TMX1**	**TMX2**	**TMX3**	**TMX4**	**TXNDC5**
AGR2																				
AGR3	65.0%																			
	(81.4%)																			
CASQ1	18.8%	17.4%																		
	(34.8%)	(40.2%)																		
CASQ2	15.3%	17.40%	68.4%																	
	(37.4%)	(34.0%)	(86.1%)																	
DNAJC10	16.2%	19.0%	16.8%	18.2%																
	(37.2%)	(35.8%)	(33.3%)	(33.3%)																
ERP27	28.2%	20.5%	17.2%	19.3%	18.6%															
	(41.0%)	(40.2%)	(34.4%)	(37.6%)	(35.8%)															
ERP29	20.6%	23.3%	18.2%	20.4%	18.1%	15.7%														
	(40.2%)	(39.5%)	(31.1%)	(33.0%)	(30.9%)	(38.9%)														
ERP44	22.2%	26.5%	19.2%	20.0%	20.4%	24.3%	25.3%													
	(39.4%)	(47.0%)	(42.9%)	(40.8%)	(30.6%)	(40.5%)	(42.0%)													
P4HB	20.1%	21.7%	21.6%	22.9%	20.2%	32.5%	24.50%	23.5%												
	(34.3%)	(29.2%)	(39.3%)	(38.3%)	(36.0%)	(54.1%)	(39.9%)	(42.9%)												
PDIA2	23.5%	20.8%	21.2%	20.6%	20.8%	28.6%	23.3.%	23.3%	47.3%											
	(35.5%)	(38.7%)	(31.8%)	(38.7%)	(32.1%)	(49.0%)	(37.2%)	(37.7%)	(64.5%)											
PDIA3	21.7%	16.9%	21.5%	18.8%	21.4%	21.8%	22.3%	23.1%	33.6%	31.1%										
	(37.5%)	(37.9%)	(33.3%)	(31.3%)	(37.5%)	(38.9%)	(34.1%)	(40.6%)	(51.0%)	(49.5%)										
PDIA4	19.80%	19.7%	18.8%	21.4%	17.5%	18.3%	29.6%	24.0%	36.8%	29.1%	41.9%									
	(31.6%)	(38.6%)	(38.2%)	(38.5%)	(30.8%)	(35.0)%	(48.1%)	(40.9%)	(55.1%)	(46.2%)	(60.2%)									
PDIA5	24.2%	22.1%	21.3%	21.7%	21.4%	19.6%	19.7%	22.6%	24.3%	23.1%	22.9%	21.2%								
	(45.1%)	(44.1%)	(35.1%)	(36.9%)	(40.0%)	(33.5%)	(33.9%)	(40.6%)	(38.3%)	(35.0%)	(37.1%)	(31.5%)								
PDIA6	27.6%	15.2%	24.0%	19.4%	32.7%	20.6%	17.0%	20.2%	23.5%	21.8%	24.2%	25.8%	28.1%							
	(35.2%)	(57.6%)	(40.6%)	(33.1%)	(48.0%)	(34.8)%	(32.2%)	(38.4%)	(31.4%)	(28.6%)	(33.4%)	(40.0%)	(43.1%)							
PDILT	23.9%	16.2%	20.1%	21.1%	20.4%	29.7%	19.1%	21.9%	31.8%	32.1%	22.1%	26.0%	19.9%	23.5%						
	(45.7%)	(35.9%)	(43.4%)	(41.8%)	(36.1%)	(54.1%)	(38.5%)	(40.9%)	(52.4%)	(51.3%)	(37.1%)	(46.7%)	(35.3%)	(36.3%)						
TMX1	18.6%	24.8%	23.7%	17.2%	21.1%	23.9%	15.5%	25.8%	29.9%	25.7%	30.0%	25.1%	35.0%	36.2%	27.0%					
	(36.1%)	(36.0%)	(37.8%)	(29.9%)	(38.2%)	(39.4%)	(32.3%)	(49.0%)	(46.9%)	(40.0%)	(50.0%)	(41.3%)	(56.4%)	(59.0%)	(39.3%)					
TMX2	23.0%	18.6%	20.2%	28.6%	23.3%	22.0%	25.6%	23.10%	25.0%	28.3%	25.8%	25.7%	16.4%	24.7%	24.7%	24.6%				
	(36.1%)	(33.1%)	(39.9%)	(44.4%)	(44.8%)	(41.5%)	(43.6%)	(38.0%)	(40.9)	(44.6%)	(36.9%)	(43.7%)	(34.9%)	(38.9%)	(42.9%)	(43.1%)				
TMX3	20.0%	19.7%	20.3%	21.8%	22.4%	18.1%	20.5%	21.4%	27.8%	21.6%	23.1%	27.1%	23.5%	45.3%	19.2%	34.2%	18.7%			
	(37.4%)	(36.5%)	(43.0%)	(43.6%)	(44.1%)	(33.3%)	(35.7%)	(34.7%)	(47.9%)	(40.3%)	(41.1%)	(42.7%)	(41.2%)	(64.2%)	(32.2%)	(50.5%)	(38.4%)			
TMX4	23.5%	25.7%	27.0%	23.1%	23.7%	28.6%	19.5%	20.1%	21.8%	24.9%	24.3%	20.5%	27.2%	24.0%	25.0%	43.6%	24.4%	22.4%		
	(39.5%)	(36.6%)	(62.2%)	(47.3%)	(39.4%)	(42.9%)	(36.0%)	(37.0%)	(34.4%)	(37.0%)	(45.7%)	(32.1%)	(41.6%)	(37.1%)	(42.5%)	(65.0%)	(42.2%)	(34.2%)		
TXNDC5	21.6%	20.9%	22.9%	20.7%	25.4%	22.0%	20.8%	33.3%	26.2%	26.9%	24.4%	23.5%	27.3%	33.0%	23.3%	24.6%	29.5%	51.4%	21.6%	
	(28.4%)	(39.5%)	(37.9%)	(34.0%)	(41.5%)	(37.9%)	(32.9%)	(50.0%)	(37.2%)	(37.5%)	(38.5%)	(34.1%)	(43.8%)	(53.0%)	(34.9%)	(33.8%)	(50.0%)	(71.6%)	(38.0%)	
TXNDC12	34.2%	38.3%	20.0%	41.2%	18.5%	20.2%	27.5%	21.1%	26.2%	26.9%	24.1%	22.9%	22.7%	26.9%	23.1%	27.7%	21.6%	31.9%	18.7%	20.1%
	(50.6%)	(53.9%)	(30.8%)	(70.6%)	(36.3%)	(30.2%)	(40.0%)	(32.2%)	(37.2%)	(37.5%)	(39.7%)	(41.2%)	(43.8%)	(37.0%)	(38.2%)	(39.8%)	(37.4%)	(48.9%)	(32.1%)	(31.5%)

Sequence identity and similarity (in parentheses) are noted.

## The human *PDI* gene family

### DNAJC10

The *DNAJC10* gene is located at Chr 2q32.1 and encodes the 793-a.a. DNAJC10 protein (also commonly known as ERdj5 or MTHr) [10]. The *DNAJC10* gene consists of 3483 bp; transcription of two splice variants has been identified due to a skipped exon, resulting in a 138-bp (46 a.a.) absence, present between nucleotides 1243 and 1244 [10]. To date, a total of four missense single-nucleotide polymorphisms (SNPs) have been reported for ERdj5, located at a.a. 76 (Asp → Asn), 347 (Leu → Ile), 414 (Tyr → Cys) and 646 (His → Gln); ERdj5 also contains one potential Asn-linked glycosylation site, present at a.a. 530. Like other *PDI* family members, the *DNAJC10* promoter region contains a putative ER stress element (ERSE) box, yielding gene induction following ER stress [11]. Expression patterns of *DNAJC10* revealed ubiquitous expression with varying intensities and high levels of expression in secretory tissues such as the pancreas and testis [10,11]. In addition to the PDI family, DNAJC10 is also a member of the ERdj family, being comprised of an unverified NH_2_-terminal signal peptide (32 a.a.), one DnaJ domain (which plays a major role in protein folding), five TRX domains (one **b** and four **a** type domains, active sites Cys-Ser-His-Cys, Cys-Pro-Pro-Cys, Cys-His-Pro-Cys, and Cys-Gly-Pro-Cys), and a COOH-terminal ER retention sequence (Lys-Asp-Glu-Leu) [10,12]. Despite the high number of TRX domains, DNAJC10 was found to possess roughly one-third the activity of P4HB and displayed no oxidase or isomerase activity [12]. These results were found to reflect an apparent redox equilibrium constant of 190 mM, roughly 100 times more reducing than that of the ER [12].

### ERP27

Currently, little is known about the precise role and function of *ERP27*. *ERP27* was discovered in 2006 following a database search for novel human PDI family members [13]. The *ERP27* gene has been mapped to Chr 12p12.3 and encodes the 273-a.a protein. The ERP27 protein contains a cleavable NH_2_-terminal signal sequence, leaving the mature protein to be 248 a.a, beginning at Glu-26 [13]. ERP27 does not contain a redox active Cys-X-X-Cys TRX motif, however, the protein does contain one **b** and one **b’** type domains and an ER retention sequence (Lys-Val-Glu-Leu) [13]. ERP27 was also found to directly interact with another PDI family member, PDIA3 [13]. Expression of ERP27 is fairly ubiquitous, with highest expression found in pancreas [13]. To date, no knockout studies have been conducted and future studies are needed to fully understand the function and role of ERP27.

### ERP29

The *ERP29* gene is located at Chr 12q24.13 and may represent a gene-duplication event with *ERP27*, given their close proximity. *ERP29* encodes a protein of 261 a.a. termed ERP29 (also known as ERP28). In a comprehensive study of the genomic organization of *ERP29*, Sargsyan et al*.* studied a 5′-flanking region consisting of ~2 kb, 3 exons, 2 introns and a 3′-flanking region of 0.31 kb [14]. ERP29 contains an NH_2_-terminal signal peptide (32 a.a.), one **a** type TRX domain and a COOH-terminal ER-retention sequence (Lys-Glu-Glu-Leu) [15]. While the presence of one **a** type domain is present, ERP29 is unique in that it does not contain an active-site motif; assignment of the TRX domain is placed strictly on sequence homology with the **a**-type TRX domain, not on the activity of the domain. ERP29 contains two potential phosphorylation sites, both located on tyrosine residues, located at a.a. 64 and 66. ER localization of ERP29 was also confirmed using immunofluorescence and subcellular fractionation [15]. ERP29 is ubiquitously expressed with high levels being found in secretory tissues as well as the prostate, pancreas, and liver [14,16]. Although *ERP29* lacks any identified ERSE in its promoter region, ERP29 is described as an ER stress-inducible protein and has been shown to co-localize with other ER stress-associated chaperones, glucose-regulated protein 78 (GRP78 or BiP) [16]. ERP29 has been postulated to play a role in the progression of tumorigenesis in mice; following implantation of both knockdown and over-expressed null ERP29 MCF-7 cells, a significant decrease in tumor size and altered morphogenesis was observed in mice [17]. Currently, no knockout mouse is available for *Erp29*.

### ERP44

The *ERP44* gene is located at Chr 9q22.33 and encodes the 406-a.a. ERP44 protein [18]. ERP44 was discovered following immunoprecipitation experiments with human endoplasmic reticulum oxidoreductase-1α (ERO1-Lα) and was originally identified as KIAA0573 [18]. The coding sequence of *ERP44* contains 12 exons and there is an ERSE in the promoter region of the gene [18]. The human ERP44 protein contains an unverified NH_2_-terminal signal peptide (29 a.a.), three TRX-like domains (one being the catalytically active **a** domain with a Cys-Arg-Phe-Ser active site), and the ER-retention sequence (Arg-Asp-Glu-Leu) [18]. The precise physiological function of ERP44 has yet to be determined; however, oxidation of ERO1α has been observed, suggesting that ERP44 may control the function of the ERO1 proteins, thus controlling the redox state of the ER [18]. ERP44 is up-regulated during ER stress response and has also been proposed to play a role in adiponectin secretion which influences glucose regulation and fatty acid catabolism [18,19].

### P4HB

*P4HB* is the first described member of the *PDI* gene family and was originally identified as the β-subunit of human prolyl-4-hydroxylase (P4H) [20,21]. The *P4HB* gene is located at Chr 17q25 and consists of 11 exons [20,22]. The promoter region of *P4HB* contains 11 protein binding sites, including an ERSE, underscoring the dynamic nature of *P4HB* transcriptional regulation [23]. Although the presence of an ERSE has been confirmed in the promoter region of the gene, P4HB is considered to be a weakly-induced ER stress protein, likely due to its high abundance. The promoter region also contains six CCAAT elements in the first 378 nt of the gene, and mutations introduced into any of these elements was found to reduce promoter activity by up to 50% [23].

The *P4HB* gene encodes a 508-a.a. protein containing a 17-a.a. signal peptide, four TRX domains with two **a** type (Cys-Gly-His-Cys, Cys-Gly-His-Cys), an Asp/Glu rich domain (a.a. 480 to 500), and a COOH-terminal ER retention sequence (Lys-Glu-Asp-Leu) [2]. P4HB is ubiquitously expressed in nearly all tissues and is very highly abundant; estimations predict P4HB to account for up to 0.8% of total cellular protein [24]. Currently, a crystal structure has yet to be resolved for the full, intact protein, although multiple domains have been solved. Despite this, P4HB remains the most widely studied and understood protein in the family. P4HB is effective at oxidizing, reducing, and isomerizing disulfide bonds both *in vitro* and *in vivo* and exists as a homodimer [25,26]. Although its role in disulfide bond generation remains the most widely studied enzymatic action, P4HB has also been shown to exhibit chaperone-like activity, demonstrating an additional role in maturation of nascent proteins regardless of the presence of disulfide bonds [27,28]. P4HB has also been shown to be an essential subunit for microsomal triglyceride transfer protein and P4H [21,29]. To date, no viable knockout mouse strain for *P4hb* has been reported, likely due to its critical role in disulfide bond generation [3].

### PDIA2

*PDIA2* was identified in 1996 as a pancreas-specific member of the PDI family, resulting in its common name, PDIp [30]. Located on Chr 16p13.3, the initial characterization of the *PDIA2* gene revealed that it encodes a protein with an ORF of 511 a.a; these studies were unable to validate an in-frame stop codon located 5′ upstream of the ATG start site [30]. Due to this discrepancy, the PDIA2 sequence was verified in 2006 to have an NH_2_-terminal signal sequence, generating a protein of 525 a.a. in length. The *PDIA2* gene also yields two splice variants encoding two isoforms of the mature PDIA2 protein, varying by three amino acids in length (isoform 2 does not contain a.a. 181 to 183). Multiple SNPs have been detected in the *PDIA2* gene, resulting in mutations at a.a. 39 (Pro → Ser), 119 (Thr → Arg), 185 (Glu → Lys), 286 (Thr → Met), 382 (Pro → Ala), 388 (Arg → Gln), and 502 (Pro → Ser).

The PDIA2 protein contains two **a** type (Cys-Gly-His-Cys and Cys-Thr-His-Cys active sites) and one **b** and one **b’** type domains; although redox active, PDIA2 was found to be less effective than P4HB in assays for both reduction and oxidation of disulfides [30]. In addition to its role as a folding catalyst, PDIA2 has been proposed to play a role in the production and secretion of digestive enzymes *in vivo*[31]. Evidence has also suggested a role for PDIA2 in the binding and regulation of intracellular 17β-estradiol levels, thus regulating estrogen synthesis [32]. PDIA2 contains three sites for Asn-linked glycosylation, located at residues 127, 284, and 516 [30]. To date, no knockout studies have been conducted with *Pdia2*.

### PDIA3

Originally identified as phospholipase C alpha, the *PDIA3*gene, located at Chr 15q15, encodes the 505-a.a PDIA3 protein (also commonly known as ERp57, ERp60, P58) [33]. Like other PDI family members, PDIA3 contains a signal peptide, corresponding to the first 24 a.a., yielding a mature protein of 481 a.a. in length [34]. PDIA3 contains two **a** type (Cys-Gly-His-Cys and Cys-Gly-His-Cys active sites), one b and one b’ type domain and an ER retention sequence, Gln-Glu-Asp-Leu [35,36]. PDIA3 expression has been detected in liver, placenta, lung, pancreas, kidney, heart, skeletal muscle, brain, and spermatozoa [33,37]. Presently, only one missense SNP has been reported for *PDIA3*, resulting in a mutation of Lys → Arg at a.a. 415.

The precise physiological role of PDIA3 has come under much scrutiny. Following the initial characterization of the protein *in vitro*, PDIA3 (termed ERp60 or P58 at the time) was identified as a cysteine protease despite little sequence homology with other heavily studied cysteine proteases [35]. In 1995, however, PDIA3 was determined to be redox active, showing the ability to reduce insulin disulfides. Bourdi et al*.* were also able to definitively prove that PDIA3 possessed no protease activity [36]. PDIA3 has also been shown to play a role in the correct folding of glycoproteins when in a complex containing both calnexin and calreticulin [38]. The physiological role of PDIA3 has also been investigated in rodent models, utilizing *Pdia3(−/−)* knockout mice. Although ubiquitous deletion of *Pdia3* was found to be embryolethal, heterozygous knockouts were generated, revealing multiple bone abnormalities, most notably in the femur [39]. Ablation of *Pdia3* was found to abolish signaling induced by 1,25-dihydroxyvitamin D_3_, a crucial regulator of bone and cartilage development, by eliminating signaling through protein kinase C [39]. Additional knockout studies in murine B cells revealed a critical role for PDIA3 in the presentation of antigens by major histocompatibility complex I molecules [40]. Although additional work is needed, these studies suggest a wide array of physiological roles for PDIA3.

### PDIA4

*PDIA4* is located at Chr 7q35 and encodes the 645-a.a. PDIA4 protein (commonly known as ERp72). Although not much is known about the physiological role of *PDIA4*, studies indicate that this gene is induced following ER stress; these results have been found to be the result of a putative ERSE in the promoter region of the gene [41,42]. Like other PDI family members, PDIA4 contains an NH_2_-terminal signal peptide of 21 a.a., yielding a mature protein of 625 a.a. [43]. PDIA4 contains five TRX domains, three **a** type (with all three active sites being comprised of Cys-Gly-His-Cys), one b and one b’ type; PDIA4 also contains the ER retention sequence (Lys-Glu-Glu-Leu) [43,44]. Mutagenesis studies to the active-site cysteines revealed varying degrees of decreased enzymatic activity, whereas mutagenesis to multiple domains revealed a more pronounced reduction in enzymatic activity of the protein [44]. PDIA4 also contains a string of highly acidic residues near the NH_2_-terminus of the protein; while the precise role of these residues remains unknown, they have been proposed to play a role in regulation of Ca^2+^, yielding its rat homolog name calcium-binding protein-2 (CaBP2) [45]. PDIA4 is a fairly ubiquitously expressed protein, although less abundant than PDI, expression patterns are similar to those of PDIA3 [46]. One missense SNP has been reported in the *PDIA4* gene, resulting in a mutation located at residue 173 (Thr → Met). Studies analyzing *Pdia3* knockdown revealed partial functional restoration by the PDIA4 protein, although, to date, no *Pdia4* knockout mouse has been generated [47].

### PDIA5

Although discovered in 1995, little is known about the precise role of the *PDIA5* gene. Located on Chr 3q21.1, *PDIA5* encodes the 519-a.a. PDIA5 (or PDI-related protein). PDIA5 contains four TRX domains (three **a** and one **b**-like domain), made up of active sites Cys-Ser-Met-Cys, Cys-Gly-His-Cys and Cys-Pro-His-Cys, a COOH-terminal ER retention sequence (Lys-Glu-Glu-Leu), and an unverified signal sequence comprising the first 21 a.a. [48]. Despite an additional Cys-X-X-Cys motif, Horibe et al*.* revealed that PDIA5 has significantly less enzymatic activity than that of P4HB [49]. The contributions of each Cys-X-X-Cys active site were also investigated, revealing varying degrees of altered activity following mutations to each, or multiple, active sites [49]. It was concluded that the second active site (Cys-Gly-His-Cys) was the most critical for isomerase activity and that all three motifs are not required for activity [49]. Much like P4HB, PDIA5 was also shown to exhibit chaperone-like activity by refolding denatured rhodenase, which does not contain any disulfide bonds [49]. PDIA5 mRNA has been detected in liver, kidney, lung, and brain––with the highest level of secretion being noted in the liver [48]. Although to date no ERSE has been identified, *PDIA5* has been shown to be moderately up-regulated following induction of the ER-stress response in cultured cells [48]. In a 2011 study by Carbone et al., a significant association was found between the SNP, rs11720822, and primary open-angle glaucoma in two separate populations [50]. No viable knockout mouse has been generated for *Pdia5*.

### PDIA6

Much like *PDIA5*, little is known about the role of *PDIA6* both *in vitro* and *in vivo.* The *PDIA6* gene is located at Chr 2p25.1 and encodes the 440-a.a. PDIA6 protein (commonly reported as P5 or ERP5). PDIA6 contains an NH_2_-terminal signal sequence of 19 a.a., three TRX domains (two **a** type and one **b**) consisting of two Cys-Gly-His-Cys active sites, an Asp/Glu rich domain and a COOH-terminal Lys-Asp-Glu-Leu ER-retention sequence [51,52]. Recombinant PDIA6 demonstrates both isomerase and chaperone activities, although approximately 45% and 50% to 60% to that of P4HB, respectively [53]. Point mutations have also been conducted to the active-site cysteines, revealing that NH_2_-terminal cysteines in each active site exhibit the majority of isomerase activity [53]. *PDIA6* contains an ERSE in its promoter region, which was recently validated *in vitro* utilizing over-expression of the ER-stress transcription factor, X-box protein-1 (XBP-1); PDIA6 was found to be significantly increased, demonstrating inducibility by the unfolded protein response [54,55]. A complete expression profile for *PDIA6* has yet to be conducted; however, high levels of the protein were detected in platelets. Cell-surface expression of PDIA6 was found to be necessary for the proper development and function of platelets, whereas inhibition of the protein using anti- PDIA6 antibodies revealed inhibition of platelet aggregation [51]. PDIA6 has also been shown to directly interact with GRP78 (or BiP) suggesting a role for PDIA6 in the refolding of substrates that have been targeted to BiP [56]. Presently, one missense SNP has been identified (rs4807) resulting in a point mutation at a.a. 214 (Lys → Arg). At present, no viable *Pdia6* knockout mouse is available.

### PDILT

Expression of the *PDILT* gene has been reported to be exclusively limited to the testis. *PDILT* is located on Chr 16p12.3, encoding the 584-a.a. PDILT protein [57]. Despite the presence of two **a** type TRX domains (with non-classical Ser-Lys-Gln-Ser and Ser-Lys-Lys-Cys motifs), PDILT does not exhibit the ability to oxidize or reduce disulfide bonds, although evidence has supported PDILT to engage in disulfide-bonded complexes *in vitro*[57,58]. PDILT contains a predicted NH_2_-terminal signal peptide of 20 a.a. in length, a COOH-terminal ER-retention sequence (Lys-Glu-Glu-Leu) and is heavily glycosylated through nine potential Asn-linked glycosylation sites [57]. Much like P4HB, PDILT also interacts with the oxidoreductase ERo1α in cultured cells, suggesting a role in the shuffling of electrons in the ER lumen [57]. No knockout mouse is currently available and considerable research is needed to fully elucidate the precise role of *PDILT*.

### TXNDC5

*TXNDC5* is located at Chr 6p24.3 and encodes the 432-a.a. endothelial PDI, TXNDC5 (or EndoPDI) protein [59]. Despite its discovery in 2003, little research has been conducted on the role of *TXNCD5 in vivo*. TXNDC5 contains a signal peptide of 32 a.a., three **a-**type TRX domains all with Cys-Gly-His-Cys active sites, and a COOH-terminal ER retention sequence, KDEL [59]. *TXNDC5* was originally identified in a screen for proteins highly expressed in endothelial cells, leading to its common name, EndoPDI. Subsequent studies revealed *TXNDC5* expression in a number of tissues with the highest expression being found in lymph nodes, stomach, pancreatic islets, and heart [59,60]. TXNDC5 is induced under conditions of hypoxia, and loss of TXNDC5 leads to an increase in apoptotic cell death in microvascular endothelial cells during hypoxia, but not normoxia [59]. Preliminary studies have also implicated a role for TXNDC5 in diabetes, noting a decrease in the expression of the protein in pancreatic islets in animals with consistently elevated glucose levels [60]. In a 2010 study, Jeong et al*.* investigated the role of *TXNDC5* in development of the skin disorder vitiligo [61]. A total of 230 Korean patients with non-segmental vitiligo were investigated for SNPs in the *TXNDC5* gene; in total, seven SNPs were identified in the *TXNDC5* gene, three of which (rs1043784, rs7764128, and rs8643) demonstrated an association with the vitiligo phenotype [61]. Although relevant *in vivo* studies have been conducted on the role of *TXNDC5* no biochemical parameters have been evaluated, with regard to its role in disulfide bond oxidation and reduction. A viable knockout mouse has not been generated for the study of *Txndc5*.

### The anterior gradient homolog genes

#### *AGR2*

The *AGR2* gene is located at Chr 7p21.3 and encodes the 175-a.a. anterior gradient protein 2 homolog (AGR2) [62,63]. The AGR2 protein has an NH_2_-terminal signal sequence of 20 a.a., one TRX domain (with active site Cys-Pro-His-Ser), and a COOH-terminal ER-retention sequence (Lys-Thr-Glu-Leu) [63]. Human AGR2 was originally identified in estrogen receptor-positive MCF7 cells using suppression subtractive hybridization [64]. Expression of *AGR2* transcripts has been detected in lung, pancreas, trachea, stomach, colon, prostate, and small intestine. *AGR2* has also been investigated as a potential biomarker for hormone-responsive breast cancer in estrogen receptor-α-positive breast cancer cell lines [64].

Utilizing knockout studies in mice, the *Agr2* gene was found to result in the inability to produce mucin, leading in an increased susceptibility to experimentally induced colitis and intestinal disease [65]. Due to its role in disulfide bond generation, it was hypothesized that AGR2 was responsible for the processing of MUC2, the major intestinal mucin. This protein contains >200 cysteine residues involved in various inter- and intra-protein disulfide bonds and has been found to directly associate with AGR2 [65]. Following this report, Zheng et al*.* investigated *AGR2* and *AGR3* as potential candidate genes for inflammatory bowel disease in humans [66]. A cohort of 2,540 patients having either ulcerative colitis or Chron’s disease was investigated for SNPs in *AGR2* and *AGR3*; in total, 30 SNPs were identified, 25 were located in the *AGR2* gene, while 5 were located in the *AGR3* gene [66]. The promoter region of the *AGR2* gene was also found to contain binding sites for hepatic nuclear factor-1, hepatocyte nuclear factor 3-α (FOXA1) and hepatocyte nuclear factor 3-β (FOXA2)––transcription factors that have been reported to play a role in the morphogenesis of goblet cell differentiation during formation. In summary, two total SNPs in the 5′ promoter region of the *AGR2* gene were found to be associated with the risk haplotype of ulcerative colitis in two independent populations, providing further evidence for a role for *AGR2* in disease pathogenesis [66].

#### *AGR3*

The *AGR3* gene is located at Chr 7p21.1 and encodes the 166-a.a. anterior gradient protein-3 (AGR3) homolog [63]. AGR3 transcripts have been detected in lung and pancreas and, resembling AGR2*,* AGR3 has been reported to be co-expressed with estrogen receptor-α-containing breast cancer cell lines, suggesting it to be a marker for hormone-responsive breast cancer [63,64]. Unlike AGR2, however, little is known about the precise physiological role of AGR3. AGR3 protein was originally identified as breast cancer membrane protein 11 (BCMP11), following a proteomic screen of membrane proteins in breast cancer cell lines, and was later named AGR3 due to the high degree of sequence homology with AGR2 (see Table 2) [67]. Like AGR2, AGR3 contains one redox-active center, comprised of amino acids Cys-Gln-Tyr-Ser, an NH_2_-terminal signal peptide composed of 21 a.a., and a COOH-terminal ER-retention sequence (Gln-Ser-Glu-Leu) [63]. Recently, an increase in AGR3 expression was observed in serous border-line ovarian tumors and low-grade serous ovarian carcinoma [68]. Utilizing Kaplan-Meier survival curves, King et al*.* also established that patients with AGR3-expressing tumors survived significantly longer than those patients lacking AGR3-expressing tumors [68].

#### *TXNDC12*

*TXNDC12*, also known as *AGR1TLP19,* and *ERP18/19*, contains conserved intron positions with respect to amino acid sequence with the *AGR2* and *AGR3* genes [63]. Persson et al*.* also reported that several of the individual exon lengths are identical (or altered with one codon) to *AGR2* and *AGR3*[63]. *TXNDC12* has been mapped to Chr 1p32.3 and contains seven exons spanning more than 35 kb [69]. *TXNDC12* is ubiquitously expressed in all tissues, with the highest expression being found in the liver and placenta. *TXNDC12* in the placenta was found to express an additional transcript of 1.2 kb which is associated with two poly(A) addition signals in its 3′-UTR [69]. The TXNDC12 protein contains 172 a.a. with 149 a.a. comprising the mature form of the protein (Ser^24^ – Leu^172^); TXNDC12 has one active site comprised of Cys-Gly-Ala-Cys and an ER-retention sequence (Glu-Asp-Glu-Leu) [70]. Unlike the other members of the AGR subfamily, extensive work has been conducted on the biochemical and physiochemical actions of TXNDC12. The enzymatic activity of TXNDC12 has been found to be limited strictly to disulfide bond generation and not reduction; these studies were also confirmed with the use of point mutations to the active-site cysteines (Cys-Gly-Ala-Cys), after which no detectable activity was found [70]. Chemical denaturation curves were also found to favor greater protein stability in the reduced form over the oxidized form, a property consistent with other PDI family members [70].

#### *The CASQ genes*

The *CASQ* genes (1 and 2) are interesting members of the *PDI* family, possessing no cysteine containing redox-active sites and therefore playing no role in the formation or reduction of disulfide bonds. As indicated, many of the *PDI* family proteins bind Ca^2+^ with relatively high capacity and low affinity [71]. Following studies on CASQ, Shin et al*.* found that the COOH-terminal Asp-rich domain played a major role in storage of Ca^2+^ through interaction with ryanodine receptor (RYR), a protein involved in Ca^2+^ release from the sarcoplasmic reticulum (SR) [72]. These proteins, therefore, possess unique functions relating to the PDI family of proteins, despite limited sequence homology.

#### *CASQ1*

The *CASQ1* gene is located on Chr 1q21 and encodes the 396-a.a. CASQ1 protein [73]. Expression of the mature CASQ1 protein is primarily limited to the SR of fast-twitch skeletal muscles [74]. Studies in rabbit have revealed CASQ1 to be a high-capacity (40 to 50 Ca^2+^ per molecule of CASQ1), moderate-affinity (Kd = 1 mM) Ca^2+^-binding protein that does not contain an ER-retention sequence [74-76]. Although experimentally unverified, the first 34 a.a. of CASQ1 encode the signal peptide, leaving the mature protein at 362 a.a. in length––which contains three TRX domains (two **b** and one **b’**) and a string of highly acidic residues from a.a. 353 – 396. Like other members of the PDI family, these Asp/Glu-rich stretches of a.a. are thought to be the primary binding regions for Ca^2+^; CASQ1 plays a major role in Ca^2+^ flux through the regulation of Ca^2+^ channel activity and interaction with Ca^2+^ directly [72]. Systemic knockout studies in mice revealed hypersensitivity to heat and volatile anesthetics, along with a phenotypic resemblance to malignant hyperthermia [77]; these effects were found to be due to increased Ca^2+^ following an increased exposure to heat [78]. CASQ1 also contains one potential Asn-linked glycosylation site, found at a.a. 350.

SNPs in the *CASQ1* gene have been reported in cases of diabetes in both Old-Order Amish and Northern European Caucasians [79,80]. Out of 26 identified SNPs, SNP CASQ-1404 (rs1186694) in the 5′ flanking region was found to have a statistically significant association with type-2 diabetes in Northern European Caucasians [79]. In a similar study analyzing type-2 diabetes susceptibility in Old-Order Amish populations, SNPs rs2275703 and rs617698 were defined as the ‘at-risk alleles’ [80]. Although mechanistically these correlations have not been confirmed, previous work has identified a putative role for Ca^2+^ release from the SR into the cytosol in regulating glucose transporter-4 (GLUT4), a key enzyme in regulation of glucose transport by insulin [80,81].

#### *CASQ2*

The *CASQ2* gene is located on Chr. 1p13.3-p11, and likely is the result of gene duplication with *CASQ1*––given their chromosomal location. *CASQ2* encodes the 399-a.a. CASQ2, which shares 91% identity with its homologue, *CASQ1* (see Table 2) [82,83] The CASQ2 protein is expressed exclusively in cardiac muscle and serves as the major Ca^2+^ reservoir in the SR of myocardium; CASQ2 also interacts with the RYR2 channel, regulating Ca^2+^ flux from the SR [76,84]. Much like CASQ1, CASQ2 contains an unverified NH_2_-terminal signal peptide (19 a.a.), three **b**-like TRX domains (two **b** and one **b’**), no ER-retention sequence, and a string of highly acidic residues (a.a. 356 to 399); CASQ2 does, however, contain one potential Asn-linked glycosylation site at a.a. 355.

The *CASQ2* gene has become a heavily researched target for diseases associated with arrhythmic heartbeats. In 2001, Lahat et al*.* investigated missense mutations found in the coding region of the gene [83]. One SNP (G −> C) was found to result in an aspartic acid changed to a histidine at a.a. 307 of the mature protein, potentially altering the Ca^2+^-chelating function of that region [83]. This SNP was found to be associated with Bedouin families from Israel susceptible to catecholine-induced polymorphic ventricular tachycardia [83]. These studies were later confirmed in *Casq2(−/−)* knockout mice, revealing susceptibility to polymorphic ventricular tachycardia following exposure to catecholamines [85].

#### *Thioredoxin-related transmembrane proteins*

The thioredoxin-related transmembrane (*TMX)* genes are newly discovered members of the *PDI* gene family. To date, little is known about the precise function of these genes; however, all four members in the PDI family consist of one TRX domain, one transmembrane domain, and non-conventional ER-retention sequences.

#### *TMX1*

Discovered in 2001, *TMX1 is* located at Chr 14q22.1 and encodes the 280-a.a. TMX1 protein [86]. TMX1 contains an NH_2_-terminal signal sequence of 26 a.a., one **a**-type TRX domain with active site Cys-Pro-Ala-Cys, one transmembrane domain (a.a. 183 to 203), and lacks an ER-retention sequence [86]. Expression of TMX1 is fairly ubiquitous, with highest levels detected in liver, kidney, placenta, and lung [86]. Mature TMX1 possesses the ability to both oxidize and reduce disulfide bonds, although chaperone-like activity has yet to be investigated [86,87]. The *TMX1* gene does not contain a putative ERSE in the promoter region, supporting evidence that *TMX1* is not induced by numerous ER-stress-inducing agents; over-expression of the protein in cultured cells, however, has revealed amelioration of both Brefeldin A-induced apoptosis and tunicamycin-induced ER stress [86,88]. No knockout mouse has been generated for the study of *Tmx1*.

#### *TMX2*

Perhaps the least researched gene in the family, *TMX2* was discovered in 2003 [89]. Located on Chr 11cen-q22.3, *TMX2* encodes the 296-a.a. TMX2 protein [89]. Like TMX1, TMX2 contains a COOH-terminal signal peptide (48 a.a.), one **a**-type TRX domain (Ser-Asn-Asp-Cys active site), one transmembrane domain (a.a. 104 – 126), and an ER-retention sequence comprised of Lys-Lys-Glu-Ile [89]. Expression of TMX2 is fairly ubiquitous––with high levels detected in heart, brain, liver, kidney, and pancreas [89]. Although Meng et al*.* provided the initial characterization of the protein, sequence discrepancies have been found. The official NCBI sequence of the TMX2 protein reveals a protein of 296 a.a and a second isoform, lacking an in-frame exon in the central coding region, encoding a protein of 258 a.a (isoform 2 differs between a.a. 84 to 122). Future studies are required to fully elucidate the role of *TMX2 in vivo.* The availability of a *TMX2* knockout mouse has not been reported.

#### *TMX3*

The human *TMX3* gene is located at Chr 18q22 and encodes the 454-a.a. TMX3 protein [90]. Following cleavage of the 24-a.a. signal peptide, the 430-a.a. mature protein consists of one **a**-type TRX domain, with the active site being comprised of Cys-Gly-His-Cys, a transmembrane domain (located at a.a. 375 to 397), and the ER-retention sequence (Lys-Lys-Lys-Asp) [90]. Uncharacteristic to most PDI family members, TMX3 contains a luminal domain with weak sequence similar to that of the CASQ proteins [90]. Although no research has been conducted on the role of this domain in activity of the protein, it has been postulated to regulate Ca^2+^ in a manner similar to that of other CASQ proteins. NCBI reports two isoforms for TMX3 (one encoding a 195-a.a. protein), though experimentally this has not been validated [90]. TMX3 has been detected in brain, testis, lung, skin, kidney, uterus, bone, stomach, liver, prostate, placenta, eye, and muscle, with highest levels detected in heart and skeletal muscle [90]. TMX3 contains two sites of Asn-linked glycosylation (a.a. 258 and 313), which have been validated *in vitro* and is not induced under conditions of ER stress [90]. Although far less efficient than P4HB, TMX3 does display the ability to oxidize disulfide bonds; this is likely due to the presence of only one Cys-Gly-His-Cys active site [90].

Although no knockout mouse has been generated for *TMX3*, studies have been conducted in mice, targeting *TMX3* transcripts using morpholinos [91]. Investigating the mechanisms behind microphthalmia in humans, a genetic disease associated with retarded growth of the eye, a 2.7-Mb deletion was found at Chr 18q22.1, leading to deletion of the *TMX3* gene [91]. Studies in mice using a targeted approach to delete the *TMX3* gene revealed a similar phenotype, which was rescued following injection of human TMX3 mRNA [91]. Sequencing of 162 patients with anopthalmia or microphthalmia revealed two missense mutations, leading to the missense SNPs, R39N, and D108N [91]. Future studies are required to fully elucidate the precise role of *TMX3*, although preliminary studies reveal exciting areas for research.

#### *TMX4*

The *TMX4* gene is located at Chr 20p12 and encodes the 349-a.a. TMX4 protein [92]. TMX4 consists of an NH_2_-terminal signal sequence (23 a.a.), one **a**-type TRX domain (a.a. 39 – 136 with the active site comprised of Cys-Pro-Ser-Cys), a transmembrane domain (a.a. 188 to 210), a string of highly acidic a.a. (224 to 334), and (like the other TMX proteins) lacks an ER-retention sequence [93]. *TMX4* is ubiquitously expressed, with highest levels detected in heart [92]. Preliminary studies show that TMX4 is not induced following conditions of ER stress and does not contain a putative ERSE in the promoter region of the gene [92]. TMX4 contains one site of Asn-linked glycosylation and two sites of Ser phosphorylation, which have all been experimentally validated [93]. Enzymatic activity was confirmed by observing reduction of insulin disulfides; a dominant-negative mutant, with the active-site cysteines mutated to serine, displayed no enzymatic activity [92]. No knockout mouse has been generated and substantial work will be required to understand the role of *TMX4 in vivo.*

## Conclusions

The PDI family of proteins consists of 21 members varying greatly in enzymatic activity, domain architecture, and tissue specificity. Although the predominant role of the PDI proteins is the regulation of protein folding *in vivo*––through the oxidation, reduction and isomerization of disulfide bonds––these proteins have also been shown to regulate calcium homeostasis in the ER and induction of the unfolded protein response (UPR). Since its discovery over 40 years ago, PDI has become one of the most highly studied proteins and, despite these advances, extensive research is still needed to fully understand the role of PDI *in vivo*. The more recently characterized *TMX* genes have displayed promise in novel therapeutics, ranging from disorders of the eye to regulation of the ER-stress response. Many of the genes in the *PDI* family contain a putative ERSE sequence in the promoter region of the gene, suggesting a role in the UPR. Further research on the role of these proteins in the UPR is required before effective therapeutics can be generated for a plethora of disease states associated with the ER-stress response. The 21 members of the PDI family of proteins encompass many physiological responses, and these proteins will likely provide compelling avenues for future research.

## Competing interests

The authors declare that they have no competing interests.

## Authors’ contributions

JJG carried out the sequence alignment and drafted the manuscript. DRP designed and funded the study. Both authors read and approved the final manuscript.
